# Loss of Cartilage Structure, Stiffness, and Frictional Properties in Mice Lacking PRG4

**DOI:** 10.1002/art.27436

**Published:** 2010-06

**Authors:** Jeffrey M Coles, Ling Zhang, Jason J Blum, Matthew L Warman, Gregory D Jay, Farshid Guilak, Stefan Zauscher

**Affiliations:** 1 Duke UniversityDurham, North Carolina; 2 Brown UniversityProvidence, Rhode Island; 3 Howard Hughes Medical Institute, and Children's Hospital BostonBoston, Massachusetts; 4 Duke University Medical Center and Duke UniversityDurham, North Carolina

## Abstract

**Objective:**

To assess the role of the glycoprotein PRG4 in joint lubrication and chondroprotection by measuring friction, stiffness, surface topography, and subsurface histology of the hip joints of *Prg4*^−/−^ and wild-type (WT) mice.

**Methods:**

Friction and elastic modulus were measured in cartilage from the femoral heads of *Prg4*^−/−^ and WT mice ages 2, 4, 10, and 16 weeks using atomic force microscopy, and the surface microstructure was imaged. Histologic sections of each femoral head were stained and graded.

**Results:**

Histologic analysis of the joints of *Prg4*^−/−^ mice showed an enlarged, fragmented surface layer of variable thickness with Safranin O–positive formations sometimes present, a roughened underlying articular cartilage surface, and a progressive loss of pericellular proteoglycans. Friction was significantly higher on cartilage of *Prg4*^−/−^ mice at age 16 weeks, but statistically significant differences in friction were not detected at younger ages. The elastic modulus of the cartilage was similar between cartilage surfaces of *Prg4*^−/−^ and WT mice at young ages, but cartilage of WT mice showed increasing stiffness with age, with significantly higher moduli than cartilage of *Prg4*^−/−^ mice at older ages.

**Conclusion:**

Deletion of the gene *Prg4* results in significant structural and biomechanical changes in the articular cartilage with age, some of which are consistent with osteoarthritic degeneration. These findings suggest that PRG4 plays a significant role in preserving normal joint structure and function.

PRG4 is expressed by synoviocytes and superficial zone chondrocytes as the glycoproteins lubricin and superficial zone protein (1–3), which contribute to boundary lubrication of loaded articular cartilage (4–6). PRG4 is also expressed in meniscus (7), tendon (8–10), ligament, heart, lung, liver, and other tissues (11,12). We use the term lubricin to collectively refer to these proteins, recognizing that variations exist in messenger RNA splicing, posttranslational modification, and cellular origins. The N- and C-terminal regions of lubricin are globular and are separated by a long, heavily glycosylated mucin-like domain (13). In addition to reducing friction, lubricin inhibits synovial cell overgrowth, reduces cell adhesion (14), and prevents cartilage–cartilage integration (15).

Osteoarthritis (OA) is a multifactorial disease with complex etiologies involving hereditary, developmental, metabolic, mechanical, and other factors. The disease progression involves a combination of abnormal mechanical stresses and biochemical imbalances that lead to a loss of proteoglycans, disruption of the collagen network, and alterations in other joint tissues such as the synovium and subchondral bone (16,17). Matrix degeneration generally begins in superficial cartilage and progresses to deeper regions of the tissue (18). Lubricin down-regulation has been seen in animal models of OA (19,20), and a low lubricin concentration has been observed in human patients following anterior cruciate ligament injury, a significant risk factor for OA (21). In recent studies, direct intraarticular injections of recombinant lubricin were shown to slow cartilage degeneration in a rat model of OA, suggesting that lubricin can play a chondroprotective role in the joint (22). A better understanding of the role of lubricin in preventing cartilage damage and degeneration may lead to improved treatment of joint diseases.

Truncating mutations in *Prg4* are responsible for the rare, autosomal-recessive camptodactyly-arthropathy–coxa vara–pericarditis syndrome (CACP) (23). Patients with this disorder have flexion contractures of phalangeal joints at an early age and develop a noninflammatory arthropathy that leads to premature joint failure (24). In addition, synovial fluid from patients with CACP shows no ability to lower friction in a latex-on-glass bearing, which can be effectively lubricated by normal synovial fluid (25). Since cartilage from CACP patients has not been available for study, cartilage from *Prg4*^−/−^ mice provides a novel experimental model for the absence of lubricin in humans. Furthermore, this cartilage also provides a convenient model substrate to investigate boundary lubrication by lubricin using atomic force microscopy (AFM). Initial studies have shown that mice lacking PRG4 exhibit changes similar to those in CACP patients, including an abnormal cartilage surface, synovial hyperplasia, and eventual joint failure (14). Cartilage surfaces of these mice were irregular, with abnormal protein deposits and a loss of superficial zone chondrocytes (14), and whole joint friction was higher than in the joints of *Prg4*^+^^/−^mice (25). Previous studies have not directly characterized the frictional or mechanical properties of the cartilage of *Prg4*^−/−^ mice due to the small size of mouse joints.

In this study, we measured the frictional properties, stiffness, and topography of cartilage of *Prg4*^−/−^ and wild-type (WT) mice ages 2–16 weeks by AFM and related these measurements to histologic appearance. The low coefficients of friction which have often been measured on cartilage are due in large part to load sharing by pressurization of the interstitial fluid, which makes up the majority of the tissue (26). Boundary lubrication can further reduce friction, and possibly wear, in regions of joint contact, acting independently of interstitial fluid pressure (27). Lubrication by interstitial fluid pressurization depends on the biphasic time constant of the tissue (28), which becomes very small for small contact areas. For microscale contacts, the time constant is on the order of milliseconds or less. Thus, AFM allows measurement of cartilage friction in near or complete absence of interstitial fluid pressurization (29,30), making it an excellent tool for the focused study of boundary lubrication properties.

## MATERIALS AND METHODS

### Lubricin-null mouse model

C57BL/6J *Prg4*^−/−^ mice and WT controls were killed by carbon dioxide asphyxiation followed by cervical dislocation at 1 of 4 ages (2, 4, 10, or 16 weeks) in accordance with protocols approved by the Institutional Animal Care and Use Committee. Limbs were then stored at –80°C until analysis. Seven *Prg4*^−/−^ mice and 7 WT mice of each age were used. Femurs were extracted with the aid of a stereomicroscope and rinsed with phosphate buffered saline (PBS) before measurements on cartilage were performed.

### Histologic analysis and scoring procedures

After AFM measurements, femurs were fixed in 10% formalin, decalcified in Cal-ex (Fisher Scientific), dehydrated in graded solutions of ethanol in water, infiltrated with xylenes, and finally infiltrated with Paraplast embedding medium (Fisher Scientific) according to a standard protocol. Sections (6-μm thick) were cut with a microtome and placed on glass microscope slides. The tissue was sliced distal to proximal, parallel to the axis of the femoral neck, and the femoral neck was used as a reference point to gauge the depth of the slices. Slices 50–70% through the femoral neck were chosen for analysis since they included the approximate region measured using AFM. Sections were stained with Harris' hematoxylin, fast green FCF, and Safranin O. Quantitative measurements of images were performed using ImageJ software (National Institutes of Health). If adequate sections were not obtained from the femur used for AFM, the opposite limb (8 data points) or, if necessary, a limb from a new mouse (1 data point) was used.

The surface of the joint typically stained for fast green FCF only, and the surface layer thickness was measured from the joint surface to the point where Safranin O staining first appeared. Uncalcified cartilage thickness was measured from the tidemark to the base of the surface layer. Both surface layer thickness and uncalcified cartilage thickness were measured at 3 points across an image of the anterior load-bearing region of the joint. Full cartilage thickness was measured along the axis of the femoral neck. Semiquantitative scores for degenerative changes were assigned based on images of 3 areas of each joint (anterior, medial, and posterior regions of load-bearing cartilage) by graders who were blinded to age and the *Prg4* genotype. We used a modification of a previously reported (31) scoring system for mouse cartilage, including additional changes such as pericellular loss of proteoglycans and surface layer morphology. Articular cartilage structure was scored from 0 to 4, surface layer morphology was scored from 0 to 3, and pericellular loss of Safranin O staining was scored from 0 to 4 (Table 1). These scores were combined for a total score of 0–11.

**Table 1:** Scoring system for qualitative analysis of histologically stained micrographs

Category	Grade
Articular cartilage structure	
Normal	0
Some surface irregularities; no fibrillation	1
Severe surface irregularities and undulation	2
Fibrillation, clefts, or cartilage loss into superficial zone	3
Fibrillation, clefts, or cartilage loss into middle zone	4
Surface layer morphology	
Smooth	0
Some small irregularities	1
Moderately roughened or enlarged	2
Severely enlarged; cellular infiltrate	3
Pericellular loss of Safranin O staining	
No pericellular loss of staining	0
Mild loss of staining around <50% of cells	1
Mild loss of staining around ≥50% of cells or moderate loss of staining around <50% of cells	2
Moderate loss of staining around ≥50% of cells or severe loss of staining around <50% of cells	3
Severe loss of staining around ≥50% of cells	4

### AFM

AFM measurements were performed with an MFP-3D atomic force microscope (Asylum Research) on the anterior region of the femoral head as we have reported previously (30). All measurements were done in PBS at room temperature. Frictional properties and compressive moduli were measured using custom AFM cantilevers with 10 μm spherical tips prepared by gluing borosilicate microspheres (Duke Scientific Corporation) to the end of triangular silicon nitride cantilevers (spring constant ≃ 0.58 N/m; Veeco). These were functionalized with octadecane thiol (SH[CH_2_]_17_CH_3_; Sigma-Aldrich), chosen for its methyl functionality as a first-order approximation of natural cartilage surface chemistry (32). Normal force spring constants were found using the MFP-3D software provided by Asylum Research (33,34), and lateral calibration constants were calculated with the wedge method (35), using a 30° silicon wedge (Advanced Scanning Probe Solutions) (36) and equations modified for spherical probes (37). A fresh probe was used for each cartilage sample.

The effective elastic modulus, an aggregate measure of stiffness at the surface of the tissue, was determined by indenting cartilage specimens to a maximum force of 100 nN with a constant velocity of 1 μm/second and fitting the approach to a Hertzian contact model for a hard sphere against an infinite plane (38), a method that has been used previously to measure stiffness of articular cartilage (29,39). Indentation depths ranged from an average of 340 nm on 10-week-old WT mouse cartilage to an average of 980 nm on 10-week-old *Prg4*^−/−^ mouse cartilage. Sixteen measurements were taken across each of three 50 × 50–μm areas on the joint and averaged. Friction measurements were performed subsequent to all stiffness measurements at the same 3 locations on each joint. Friction was measured at 16 scan lines across each 50 × 50–μm area and repeated with normal loads ranging incrementally from 20 nN to 100 nN (20, 40, 60, 80, and 100 nN), alternating between increasing and decreasing load. The coefficient of friction at each location was defined as the slope of friction force versus normal force, and the mean value was recorded as the coefficient of friction for the joint.

Subsequent to stiffness and friction testing, surfaces were imaged using softer AFM cantilevers with sharp tips (spring constant ≃ 0.12 N/m, nominal tip radius 20 nm; Veeco). These cantilevers were gold coated and functionalized with triethylene glycol (SH[CH_2_]_11_[OCH_2_CH_2_]_3_OH; Sigma- Aldrich) to minimize adhesion of cartilage material to the probe. Images were obtained in contact mode, and contact forces were kept low (≤2 nN) to minimize distortion of the tissue due to the contact force. Root mean square roughness was calculated from these images after a second-order flattening procedure to remove the effects of the curvature of the femoral head.

### Statistical analysis

AFM data and semiquantitative measurements of histologic images were analyzed by Student's *t*-test, and histologic scores were analyzed by the Mann-Whitney U test to compare between genotypes at each age. Trends of histologic scores with respect to age were tested by Spearman's rank correlation. The proportions of joints exhibiting distinct abnormal features were compared by Fisher's exact test, with Bonferroni correction where applicable. *P* values less than or equal to 0.05 were considered significant.

## RESULTS

### Histologic analysis and scoring

**Figure 1:**
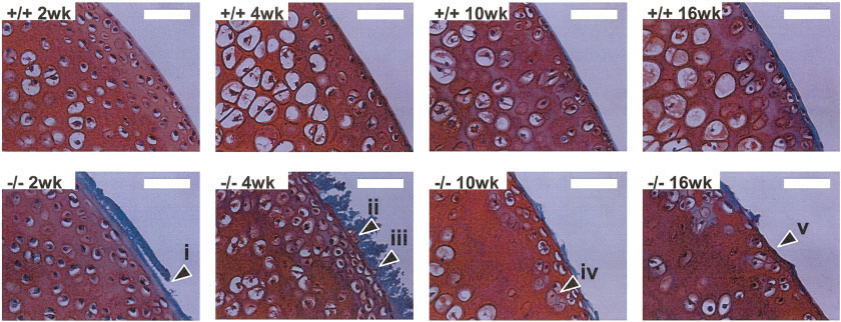
Sample micrographs of histologically stained cross-sections of cartilage from the femoral heads of wild-type and *Prg4*^−/−^ mice ages 2, 4, 10, and 16 weeks. **Arrowheads** indicate delamination of the surface layer **(i)**, a Safranin O–positive feature in the surface layer **(ii)**, a cell in the surface layer **(iii)**, pericellular loss of Safranin O staining **(iv)**, and absence of the surface layer, leaving the underlying cartilage exposed to damage **(v)**. Bars = 50 μm.

Histologic assessment of *Prg4*^−/−^ mouse cartilage revealed both surface and subsurface abnormalities (Figure 1). The surface layer and underlying cartilage surface of *Prg4*^−/−^ mouse cartilage were highly irregular relative to those of WT mouse cartilage for all ages measured, with no detectable effect of age (Table 2). Pericellular proteoglycan loss increased significantly as a function of age and was significantly higher in *Prg4*^−/−^ mouse joints at 16 weeks.

**Table 2:** Summary of qualitative grading of histologically stained micrographs*

Parameter, age	WT mice	*Prg4*^−/−^ mice
Articular cartilage structure		
2 weeks	0.64 ± 0.63	1.75 ± 1.27†
4 weeks	0.46 ± 0.34	1.75 ± 0.87†
10 weeks	0.46 ± 0.27	1.71 ± 0.34†
16 weeks	0.36 ± 0.32	1.75 ± 0.74†
Surface layer morphology		
2 weeks	0.07 ± 0.12	1.71 ± 0.62†
4 weeks	0.39 ± 0.32	2.04 ± 0.53†
10 weeks	0.14 ± 0.28	1.93 ± 0.55†
16 weeks	0.18 ± 0.19	1.68 ± 0.43†
Pericellular loss of Safranin O staining		
2 weeks	0.29 ± 0.62	0 ± 0
4 weeks	0.62 ± 0.59	1.52 ± 1.82
10 weeks	2 ± 0.69	2.62 ± 1.27
16 weeks	1.1 ± 1.44	3.05 ± 0.68†

* Values are the mean ± SD. For each age group, femoral heads from 7 mice of each genotype were examined.

† *P* < 0.05 versus wild-type (WT) mice.

**Figure 2:**
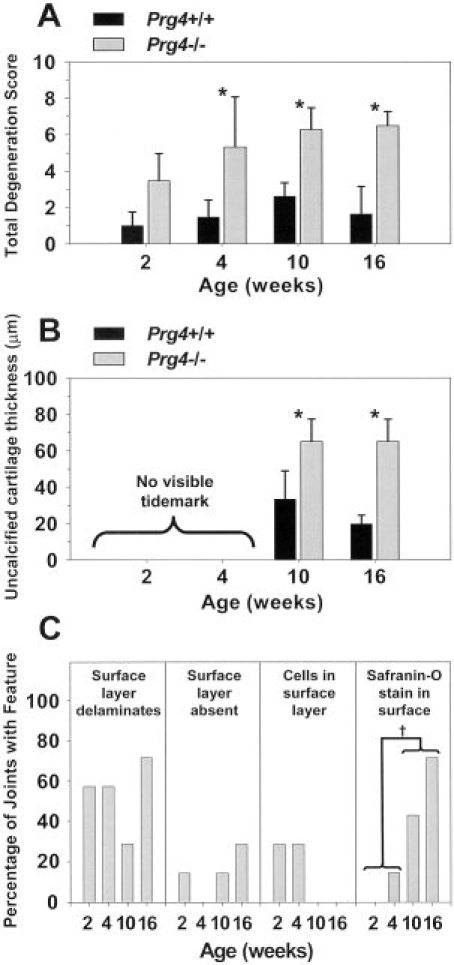
Graphs depicting qualitative and quantitative histology scores in wild-type (WT) and *Prg4*^−/−^ mice. Seven mice of each genotype were studied per age group. **A,** Total degeneration score, an aggregate score of articular cartilage structure, surface layer morphology, and loss of Safranin O staining, at each age tested. **B,** Thickness of uncalcified cartilage at ages 10 and 16 weeks. Values in **A** and **B** are the mean and SD. ∗ = *P* < 0.05. **C,** Percentages of joints of *Prg4*^−/−^ mice (joints of 7 mice at each age; only 1 joint was tested per mouse) exhibiting distinct characteristics. None of these features were observed in joints of WT mice. † = *P* < 0.05.

Overall degeneration scores were significantly higher for *Prg4*^−/−^ mouse joints than for WT mouse joints for all animals age >2 weeks (Figure 2A). These trends did not appear to vary significantly as a function of the location on the joint. The mean ± SD thickness of the surface layer averaged 4.2 ± 3.5 μm in *Prg4*^−/−^ mice and 2.4 ± 0.8 μm in WT mice and did not vary as a function of age. Measurements of uncalcified cartilage depth in joints of 10- and 16-week-old WT and *Prg4*^−/−^ mice showed that the natural process of cartilage mineralization was slowed (40) (Figure 2B). Joints of younger mice were not included in this analysis since most did not have visible tidemarks. Although full cartilage thickness was also measured from the micrographs, no thickness differences were resolved between joints of WT and *Prg4*^−/−^ mice. The enlarged surface layer of *Prg4*^−/−^ mouse cartilage was seen to be delaminated in some places and was completely absent in other areas, giving way to an underlying cartilage surface that appeared abnormally rough (Figure 1). Cells were seen within the surface layer in 4 cases, and Safranin O–positive features were also observed on *Prg4*^−/−^ mouse cartilage in 9 cases (Figure 2C), suggesting the presence of glycosaminoglycans (GAGs). Safranin O–positive features were typically present over ≤5% of the joint surface, but in 1 case were present over ∼20% of the linear surface analyzed. None of these features was observed on WT mouse joints.

Fisher's exact test with a Bonferroni correction was used to compare the incidence of each of these features with respect to genotype and to compare rates of occurrence on younger (age 2–4 weeks) and older (age 10–16 weeks) *Prg4*^−/−^ mice. Surface layer delamination and Safranin O–positive surface features were shown to occur at a significantly higher rate on *Prg4*^−/−^mouse joints than on WT mouse joints, and Safranin O–positive surface features were found to be significantly more likely in older mice.

### AFM findings

**Figure 3:**
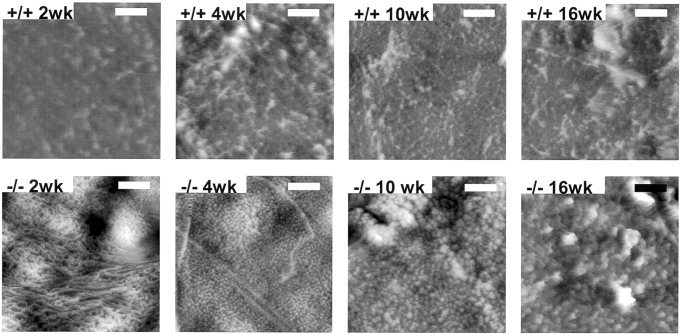
Representative atomic force microscopy images of cartilage from the anterior femoral head of wild-type and *Prg4*^−/−^ mice ages 2, 4, 10, and 16 weeks. Bars = 10 μm.

**Figure 4:**
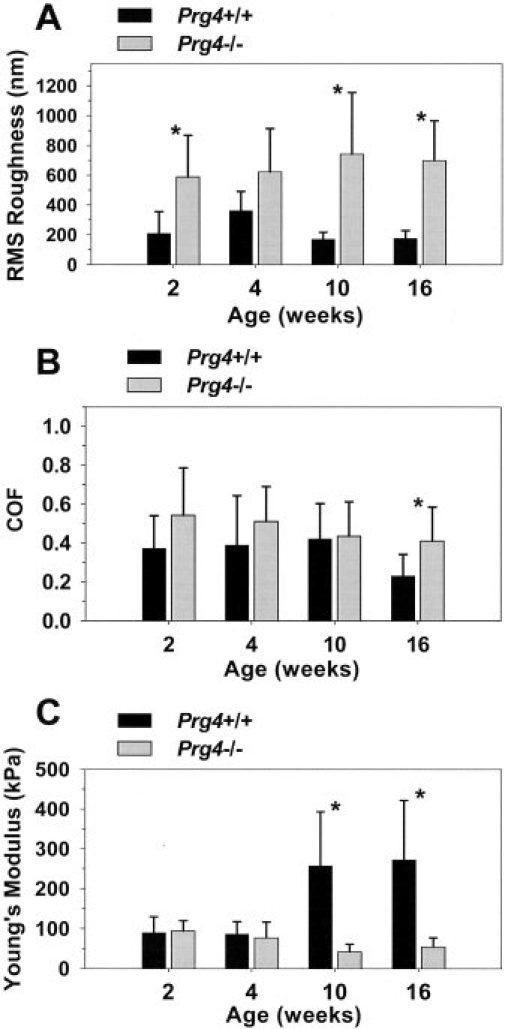
Cartilage surface properties determined by atomic force microscopy (AFM). Seven mice of each genotype were studied per age group. **A,** Root mean square (RMS) roughness measured from AFM images. **B,** Coefficient of friction (COF) between the cartilage and the functionalized probe. **C,** Effective elastic modulus of superficial cartilage. Values are the mean and SD. ∗ = *P* < 0.05.

AFM imaging allowed surfaces to be visualized at higher resolution (Figure 3). *Prg4*^−/−^ mouse joint surfaces showed increased surface roughness compared with WT mouse joint surfaces (Figure 4A) and generally were characterized by large numbers of rounded features on the scale of ∼1 μm. Meanwhile, WT mouse joint surfaces had more linear surface features. Large collagen bundles were visible in 4 of 7 AFM images of joints of 2-week-old *Prg4*^−/−^mice and in 2 of 7 AFM images of joints of 4-week-old *Prg4*^−/−^ mice. A structure similar to these thick fibrillar bundles was only observed on the joint surface of one WT mouse (age 16 weeks). The appearance of such structures predominantly on joint surfaces of young *Prg4*^−/−^ mice may relate to the reordering of the collagen fibrillar structure of *Prg4*^−/−^ mice ages 0–2 weeks that was previously observed using transmission electron microscopy (25).

The coefficient of friction was slightly higher on the joints of *Prg4*^−/−^ mice than on the joints of WT mice at age 16 weeks and was similar at younger ages (Figure 4B). The stiffness of joint surfaces of WT mice increased significantly with age, a trend that was not observed on joint surfaces of *Prg4*^−/−^ mice (Figure 4C).

## DISCUSSION

The findings of this study indicate that the presence of lubricin plays an important role in preserving natural joint structure and functional properties. *Prg4*^−/−^ mice exhibited significant changes in articular cartilage properties, including enlargement and roughening of the surface layer, irregularities in cartilage structure, and age-related changes in the compressive modulus. Cartilage of *Prg4*^−/−^ mice was also characterized by a pericellular loss of proteoglycans and delayed tidemark progression. This degenerative phenotype caused by the lack of PRG4 is similar to that observed clinically with CACP, and many of these changes are also similar to OA degeneration.

The measurements performed here show a limited effect of lubricin on frictional properties and suggest that the lubricin present on the surface of the cartilage of WT mice does not provide significant boundary lubrication. Lubricin is present in both synovial fluid and superficial cartilage, and lubricin in solution has been shown to effectively decrease the coefficient of friction of both cartilage and nonbiologic bearings in previous studies (4,6,41). The effect of lubricin on the frictional properties of the cartilage surface itself, however, has not been shown consistently in previous studies. For example, removing the surface layer by rubbing the cartilage surface with a cotton plug soaked in sodium dodecyl sulfate (42), rubbing the surface layer away by prolonged sliding (43), and cutting off the upper surface of cartilage (44) all did not result in a detectable increase in the coefficient of friction under boundary lubricating conditions. However, there is evidence that the frictional properties of the cartilage surface are influenced by levels of PRG4 expression (45,46). Removal of lubricin from the cartilage surface by NaCl extraction increased the frictional properties by only ∼20% in a system in which whole synovial fluid reduced friction by a factor of 3 relative to PBS (47).

It should be noted that the measurements performed in the present study were of the effect of a chronic rather than of a sudden absence of lubricin. Friction on these joint surfaces was affected by both the presence or absence of lubricin and by degenerative and/or compensatory changes that had occurred in the joint, as seen using AFM and histology. The structural and morphologic changes that occurred in *Prg4*^−/−^ mouse joints may have had the general effect of increasing friction, resulting in a significant difference in friction only at age 16 weeks when the most damage had occurred to the joints.

Whole joint friction measurements *Prg4*^−/−^ mouse joints have also been performed, with the finding of moderately higher friction than in joints of heterozygous mice (25). Friction coefficients measured on the cartilage surface in our study are >2 orders of magnitude higher than those shown in whole joint tests (25), a difference we attribute primarily to the presence of interstitial fluid pressurization in the joints. Although interstitial fluid pressurization provides a significantly greater influence on the frictional properties of the joint than does boundary lubrication by lubricin, it is clear that the absence of lubricin results in significant joint abnormalities and degenerative changes. These findings suggest that relatively small changes in boundary friction may have significant effects on cartilage physiology over the long term. While increased shear loading of cartilage promotes expression of various cartilage constituents, including collagen, aggrecan, and lubricin (48,49), it can also have deleterious effects, such as an increase in the production of oxidants which contribute to cartilage degeneration (50).

Alternatively, the influence of lubricin on joint health may be through mechanisms that do not involve friction directly. There is evidence that lubricin dissipates strain energy within synovial fluid in addition to lubricating joint surfaces (51). It further functions as an antiadhesive, preventing cartilage integrative repair in vitro (15). Lubricin's ability to form a surface covering, as it does on surfaces with a wide range of chemical functionalities (52,53), may provide an important property for normal joint function by conferring wear resistance in addition to boundary lubrication. We observed that the surface layer was smooth and continuous on WT mouse cartilage but was highly variable, sometimes delaminated, and occasionally absent on *Prg4*^−/−^ mouse cartilage. With a superficial surface coating, high forces occurring at a single point on the surface will be dissipated, reducing the pressure exerted at any single point on the underlying surface. The formation of lubricin dimers (54,55) and possibly further networked structures (25) likely contributes to this dissipative mechanism. The occurrence of microstructural damage in the lubricin layer rather than in the collagen proteoglycan matrix would be beneficial for 2 reasons. First, lubricin can be readily replenished but cartilage does not repair quickly, if at all (56). Second, damage to the cartilage matrix may trigger chondrocytes to produce catabolic cytokines and matrix-degrading proteases as in early-stage OA (57).

We believe the surface layer observed in histology images of WT mouse joints to be the lamina splendens, also called the superficial surface layer or surface amorphous layer, a proteinaceous, acellular, and nonfibrillar layer hundreds of nanometers to microns thick (58). Because the protein lubricin is concentrated at the cartilage surface, it is believed by many to be a major component of the lamina splendens, although direct chemical assessment of the lamina splendens has not yet been possible. Joint surfaces of *Prg4*^−/−^ mice showed significant accumulation of surface material beyond that seen on joint surfaces of WT mice, likely composed of adsorbed proteins from synovial fluid (14). The surface layer on joints of *Prg4*^−/−^ mice sometimes contained cells and localized Safranin O–positive areas, features that were not seen in WT mice. The source of the Safranin O–positive formations seen within the surface layer is unclear, but these may have been metabolized proteoglycans from within the cartilage matrix (59).

The cartilage stiffness measured at the microscale represents an aggregate stiffness of the surface layer and superficial zone cartilage. Using average measurements of surface layer thicknesses from our histologic analysis and assuming that the surface layer is softer than the underlying cartilage, we conclude from the analysis of Perriot and Barthel (60) that stiffness values are typically dominated by the surface layer and somewhat influenced by the underlying tissue stiffness of joints of both WT and *Prg4*^−/−^ mice. The effect of the underlying surface on stiffness values is smaller on joints of *Prg4*^−/−^ mice because the surface layer is thicker. The proteoglycan loss in joints of *Prg4*^−/−^ mice was predominantly localized in the pericellular region, suggesting that it was mediated primarily by the chondrocytes. The mechanism of proteoglycan loss remains to be determined but most likely involves the up-regulation of matrix metalloproteinases and aggrecanases in response to both mechanical and biochemical factors (61–63). Our observations are intriguing, since the loss of cartilage stiffness in the superficial zone is one of the earliest signs of cartilage damage in early-stage OA (18,64,65).

Lubricin appears to have a limited role in reducing the coefficient of friction on the cartilage surface of mammalian joints when only present on the articular surface. Despite this, it is apparently necessary for preservation of surface integrity, superficial stiffness, and GAG content. Further exploration of the mechanisms by which lubricin protects joints may lead to improved treatments for OA.
